# Promoter hypermethylation inactivates CDKN2A, CDKN2B and RASSF1A genes in sporadic parathyroid adenomas

**DOI:** 10.1038/s41598-017-03143-8

**Published:** 2017-06-09

**Authors:** Ashutosh Kumar Arya, Sanjay Kumar Bhadada, Priyanka Singh, Naresh Sachdeva, Uma Nahar Saikia, Divya Dahiya, Arunanshu Behera, Anil Bhansali, Sudhaker D. Rao

**Affiliations:** 1 Department of Endocrinology, Postgraduate Institute of Medical Education & Research (PGIMER), Chandigarh, India; 2 Department of Histopathology, Postgraduate Institute of Medical Education & Research (PGIMER), Chandigarh, India; 3 Department of General Surgery, Postgraduate Institute of Medical Education & Research (PGIMER), Chandigarh, India; 4 Bone & Mineral Research Laboratory, Henry Ford Hospital, Detroit, USA

## Abstract

Cyclin D1, a G1-S phase regulator, is upregulated in parathyroid adenomas. Since cyclin-dependent kinase (CDK) inhibitors, CDKN2A and CDKN2B, and RASSF1A (Ras-association domain family 1, isoform A) are involved in G1-S phase arrest and act as potential tumor suppressor genes, we aimed to study potential methylation-mediated inactivation of these genes in parathyroid adenomas. Gene expressions of cyclin D1 (CCND1) and regulatory molecules (CDKN2A, CDKN2B and RASSF1A) was analysed in parathyroid adenoma tissues (n = 30). DNA promoter methylation of cyclin D1 regulators were assessed and correlated with clinicopathological features of the patients. Gene expression analysis showed a relative fold reductions of 0.35 for CDKN2A (p = 0.01), 0.45 for CDKN2B (P = 0.02), and 0.39 for RASSF1A (p < 0.01) in adenomatous compared to normal parathyroid tissue. There was an inverse relationship between the expressions of CDKN2A and CDKN2B with CCND1. In addition, the promoter regions of CDKN2A, CDKN2B, and of RASSF1A were significantly hyper-methylated in 50% (n = 15), 47% (n = 14), and 90% (n = 27) of adenomas respectively. In contrast, no such aberrant methylation of these genes was observed in normal parathyroid tissue. So, promoter hypermethylation is associated with down-regulation of CCND1 regulatory genes in sporadic parathyroid adenomas. This dysregulated cell cycle mechanism may contribute to parathyroid tumorigenesis.

## Introduction

Primary Hyperparathyroidism (PHPT) is characterized by hypercalcemia associated with elevated or non-suppressed serum parathyroid hormone (PTH) levels^1^. The underlying molecular mechanisms for development of parathyroid adenoma are not completely understood, but the role of Cyclin D1 (encoded by the CCND1 gene) in parathyroid tumorigenesis is well established. Cyclin D1, a cell cycle regulator responsible for G1-S phase transition, is overexpressed in 20–40% of parathyroid adenomas^2–4^, although a much higher proportion (~80%) of sporadic parathyroid adenomas from Asian Indians overexpress Cyclin D1^5^. Studies in transgenic mice have confirmed that overexpression of cyclin D1 due to PTH-CCND1 rearrangement can lead to parathyroid gland growth and adenoma formation^6, 7^.

During progression of cell cycle, cyclin D1 binds to cyclin dependent kinases (CDKs) particularly CDK4 and CDK6. These kinases compete with the CDK inhibitors, p16 and p15, and block the binding of CDKs to cyclin D1 resulting in G1 phase arrest^8^. p16 and p15 proteins are encoded respectively by CDKN2A and CDKN2B, and act as tumor suppressor genes^9, 10^. Reduced expression of these inhibitor proteins in parathyroid adenomas does not appear to be due to either deletion or mutations in these genes^11^, but epigenetic changes, such as DNA methylation, could have a major role in the transcriptional silencing of gene expression. DNA methylation is the most widely studied epigenetic alteration, but there are only a few such studies in parathyroid adenomas^12–17^. In one study, there was very low level of methylation in the CDKN2A without an aberrant methylation in the CDKN2B^13^, and another study showed promoter methylation mediated silencing of both CDKN2A and CDKN2B genes^14^. Hypermethylation in CDKN2A has been reported in many other cancers but hypermethylation of CDKN2B is not as frequent^18, 19^. Ras-association domain family 1, isoform A (RASSF1A), a Ras binding protein, is another molecule that blocks G1-S phase transition and inhibits cyclin D1 accumulation^20^. Reduced expression of RASSF1A and promoter DNA hypermethylation have been reported in parathyroid adenomas^12, 13^. However, there is no clear association between the promoter DNA hypermethylation of these G1-S phase regulating tumor suppressor genes and the clinico-pathological features of parathyroid adenoma.

In the present study we analysed the expression patterns of three potential tumor suppressor genes, CDKN2A, CDKN2B and RASSF1A, in sporadic parathyroid adenomas. We further examined the methylation status of CpG sites in promoter regions of these genes as a potential epigenetic modification to explain their reduced expression patterns.

## Results

### Characteristics of PHPT patients

A total of 30 PHPT patients (9 men, 21 women) with a mean age of 40.8 (range 16–65) years were recruited. Relevant clinical and biochemical data are summarized in Table 1. Bone pain was the most common presentation (25/30 or 83%) followed by weakness and fatigue (53%), fractures (37%), nephrolithiasis (33%), weight loss (27%), cholelithiasis (20%) and pancreatitis (10%). Twenty-one (70%) patients were vitamin D deficient defined as serum 25-hydroxyvitamin D level <20 ng/ml, and the mean parathyroid adenoma weight was 4.1 g (range 0.26–25 g).

**Table 1:** Base line characteristics of PHPT patients.

Variable	Number or mean ± SD (range)
Subjects	30
Women/Men	21/9
Age (y; mean ± SD)	40.8 ± 10
Calcium (8.6–10.2 mg/dl)	11.7 ± 1.5 (8.97–15.3)
Inorganic Phosphate (2.4–4 mg/dl)	2.4 ± 0.5 (1.61–3.7)
Alkaline Phosphatase (40–129 U/L)	319.7 ± 217.1 (73–947) (Geometric mean, 256)*
iPTH (15–65 pg/ml)	732.5 ± 711.7 (73–3726) (Geometric mean, 484.5)*
25 (OH) D (11–42.9 ng/ml)	18.9 ± 14.3 (3.7–61.43) (Geometric mean, 13.23)*
Creatinine (0.5–1.2 mg/dl)	1.0 ± 0.6 (0.3–2.6)
Adenoma weight (g)	4.1 ± 5.4 (0.26–25) (Geometric mean, 2.42)*

*The data are non-normally distributed

### Gene Expression analysis

**Figure 1:**
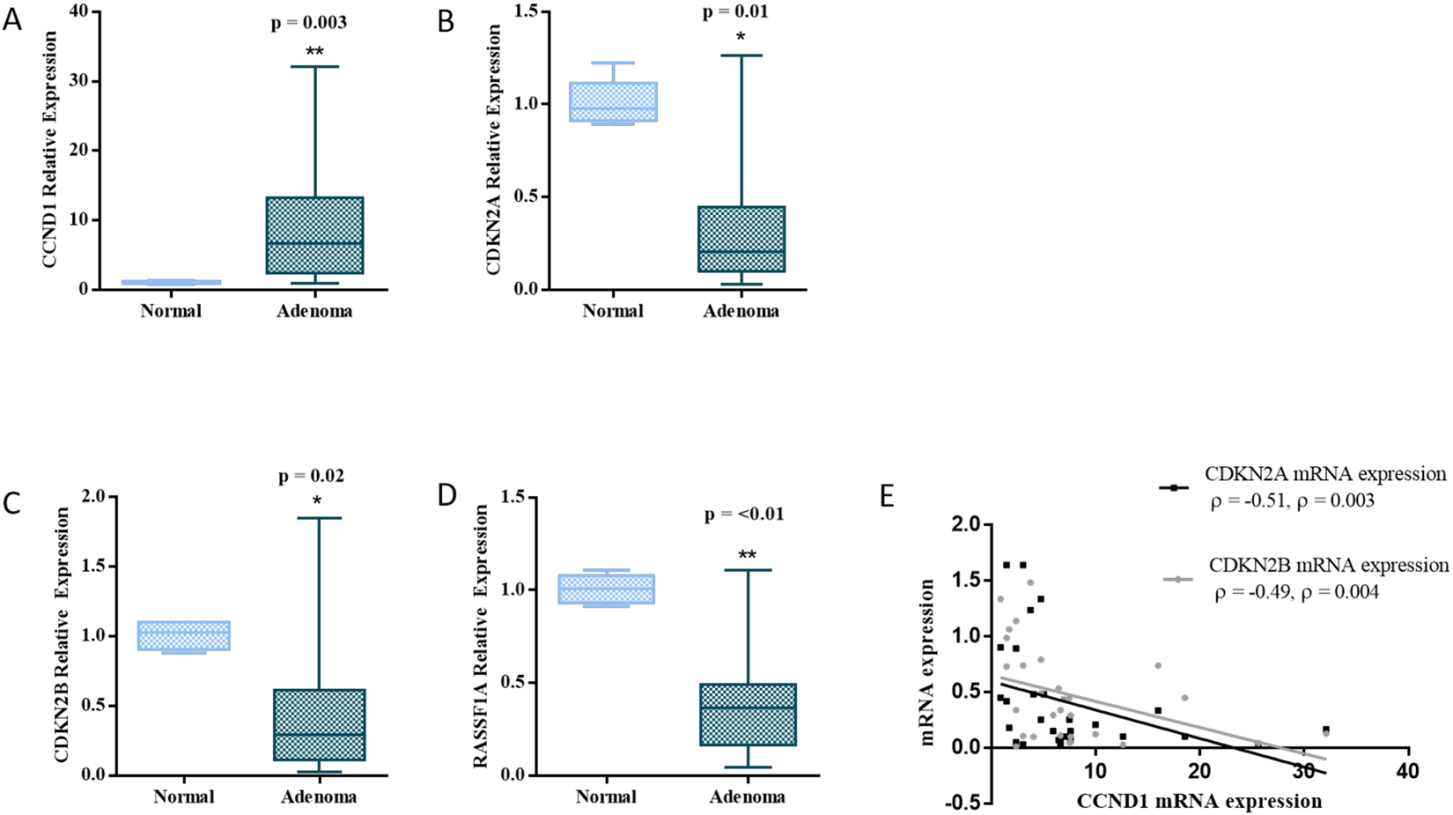
Gene expression pattern of CCND1, CDKN2A, CDKN2B and RASSF1A and association of inhibitors with CCND1 expression. Box and whisker plot representing the relative expression pattern of the genes (**A**) CCND1, (**B**) CDKN2A, (**C**) CDKN2B and (**D**) RASSFIA in parathyroid adenoma samples compared to the normal parathyroid samples with bar as minimum and maximum value. (**E**) Scatter plot showing the negative association between gene expression pattern of CDKN2A, as well as CDKN2B with Cyclin D1 expression (n = 30). (*P < 0.05, **P < 0.01).

The relative expressions of the CDKN2A, CDKN2B and RASSF1A genes were analysed by quantitative RT-PCR and were related to the cyclin D1 (CCND1) expression in parathyroid adenoma samples. Cyclin D1 was over-expressed in 93% (n = 28) cases with mean fold increase of 9.0 ± 8.7 (median = 6.6, 0.86–32.13) and was significantly higher in adenomatous compared to normal parathyroid tissue (p = 0.003; Fig. 1A).

The quantitative RT-PCR analysis showed reduced CDKN2A gene expression in 87% (n = 26) of patients with a mean fold reduction of 0.35 ± 0.39 (median = 0.21, 0.03–1.26) (p = 0.01; Fig. 1B) compared to normal parathyroid samples. The CDKN2B gene expression was reduced in 83% (n = 25) of cases with a mean fold reduction of 0.45 ± 0.38 (median = 0.29, 0.026–1.85) (p = 0.02; Fig. 1C) compared to the normal parathyroid tissue. Correlation analysis showed that the expression of both CDKN2A (ρ = −0.51; ρ = 0.003) and CDKN2B (ρ = −0.49; ρ = 0.004) were inversely related to cyclin D1 expression (Fig. 1E). Although there was no significant relationship between CDKN2A expression level and biochemical indices of the disease, CDKN2B was correlated both with serum PTH level (ρ = 0.73, p = 0.001) and tumor weight (ρ = 0.65; p = 0.006). Also, serum PTH level correlated directly with parathyroid adenoma weight (ρ = 0.77; p < 0.0001). Collectively, these findings suggest that reduced CDKN2B expression was associated with the severity of PHPT as reflected by the adenoma weight and serum PTH levels.

The RASSF1A gene expression was also reduced in 90% (n = 27) of adenomatous tissues with a mean fold reduction of 0.39 ± 0.37 (median = 0.36, 0.04–0.86; p < 0.01; Fig. 1D) compared to the normal parathyroid tissue samples. However, there was no association between cyclin D1 and RASSF1A gene expression patterns (ρ = 0.1; p = 0.61). RASSF1A expression was negatively associated with the serum calcium level of the patients (ρ = −0.58, p = 0.004). Correlation analysis of expression of all the genes with the disease parameters were summarized in the Table 2 and Supplementary Figure S1.

**Table 2:** Detailed correlation analysis between gene expression and disease parameters.

	CDKN2A Expression	CDKN2B Expression	RASSF1A Expression
Calcium	−0.12 (p = 0.51)	−0.27 (p = 0.15)	**−0.58 (p** = **0.004)**
PTH	−0.01 (p = 0.95)	**0.73 (p** = **0.001)**	−0.1 (p = 0.6)
Phosphorus	−0.082 (p = 0.72)	−0.29 (p = 0.21)	0.21 (p = 0.34)
ALP	−0.07 (p = 0.75)	**0.64 (p** = **0.001)**	−0.07 (p = 0.75)
25(OH)D	−0.07 (p = 0.72)	**−0.49 (p** = **0.016)**	0.1 (p = 0.63)
Tumor weight	0.18 (p = 0.41)	**0.65 (p** = **0.006)**	−0.19 (p = 0.37)
Age	−0.17 (p = 0.38)	−0.26 (p = 0.20)	0.03 (p = 0.89)
Gender	0.28 (p = 0.14)	−0.22 (p = 0.25)	−0.22 (p = 0.23)

### Promoter region methylation of CDKN2A, CDKN2B and RASSF1A

To further understand the reason for under-expression of the genes, we analysed methylation status of the promoter regions of CDKN2A, CDKN2B and RASSF1A genes. A methylation density of >10% was considered as methylated for the specific gene studied^12^. For the CDKN2A gene, the promoter region of exon1B (nucleotide −367 to −28) was analysed. The region contains 16 CpG sites with a product size of 340 bp. Sequencing after BSP revealed that only 50% (n = 15) of adenoma samples were methylated with a mean methylation density of 24 ± 13.2% (12.5–61.5%). Nine adenomas had methylation densities of <10% and in 6 adenomas no methylation was observed at any of the CpG sites. In contrast, none of the normal parathyroid tissue samples showed methylation except a single sample showed methylation density of 7% at one CpG site. Correlation analysis revealed that promoter methylation of CDKN2A was inversely related to adenoma weight (ρ = −0.48; p = 0.046), but not with clinico-pathological features of the patients.

For CDKN2B, the promoter region (nucleotide −289 to +10) contained 18 CpG sites with a product size of 300 bp was analyzed. Sequencing analysis revealed that 47% (n = 14) of the adenomas were hypermethylated (>10% methylation density) in the CDKN2B promoter region with a mean methylation density of 24.8 ± 12% (11.5–58.3%). Eight adenomas had methylation densities of <10% and the remaining 8 adenomas had no methylation at any CpG sites. Again, none of the normal parathyroid samples had >10% methylation, although 2 normal parathyroid tissue samples were methylated (7% density) at one CpG site. CDKN2B promoter region methylation was not associated with any clinico-pathological features of the PHPT patients.

**Figure 2:**
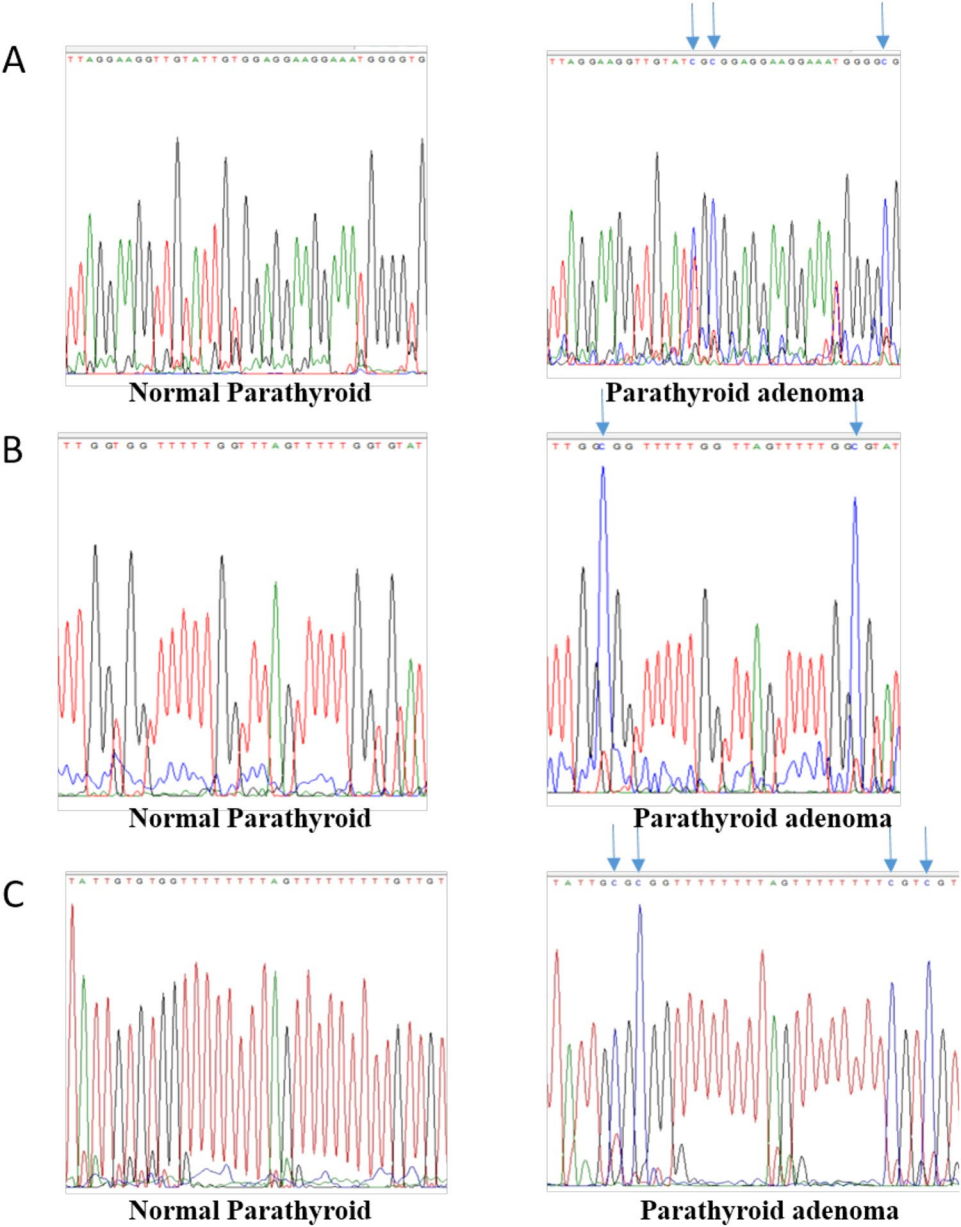
Representative electropherograms of direct sequencing of genes amplified after the bisulfite conversion of the genomic DNA samples. Representing the methylated and un-methylated cytosine of CpG sites in the promoter region of the (**A**) CDKN2A, (**B**) CDKN2B and (**C**) RASSF1A genes for both adenoma and normal parathyroid samples.

**Figure 3:**
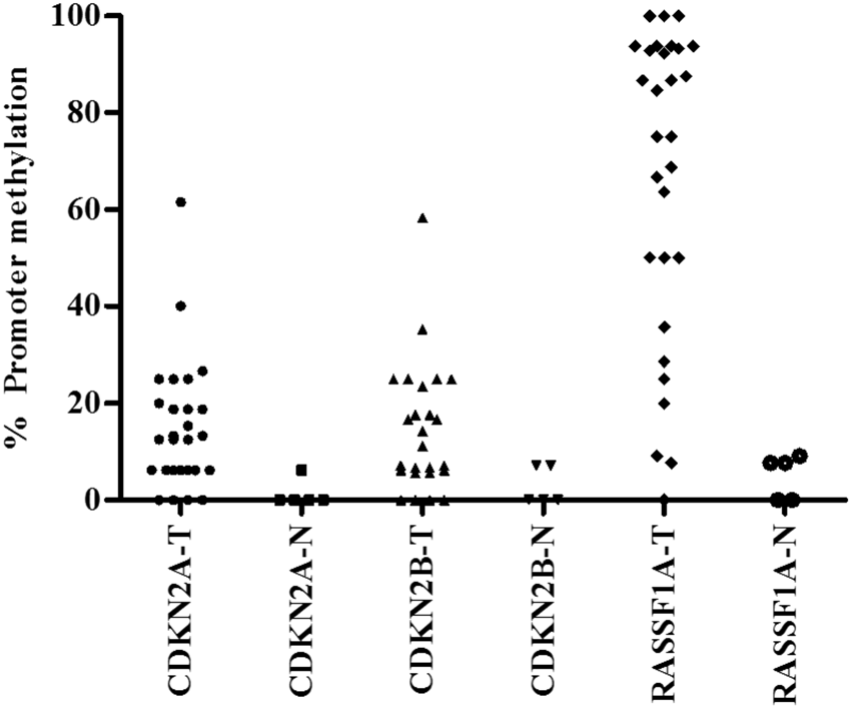
Individual value scatter plot for normal and adenoma parathyroid samples for representing promoter methylation for all the genes.

The promoter region of RASSF1A (nucleotide −145 to +55) contained 16 CpG sites and the amplified product size was 200 bp. Sequencing results revealed that RASSF1A was highly methylated (90%; n = 27) with a mean methylation density of 73.7 ± 24.9% (20–100%), and 77% (N = 23) of the adenomas were methylated at ≥50% of the CpG sites; 2 adenomas had a methylation density of 8% and 9% and one adenoma sample was not methylated at any site. As was the case with CDKN2A and CDKN2B, none of the normal parathyroid samples showed methylation of RASSF1A, although 3 of the 5 normal parathyroid tissue samples displayed low methylation densities of 7.7%, 7.7% and 9.1%. RASSF1A gene promoter methylation was inversely related to serum calcium level (ρ = −0.54, p = 0.02), suggesting that patients with severe hypercalcemia had lower level RASSF1A-DNA methylation. A similar finding was reported by Juhlin et.al.^12^. RASSF1A gene promoter methylation was not associated with PTH (ρ = −0.15, p = 0.5) and tumor weight (ρ = −0.19, p = 0.4). No other biochemical parameters were correlated with promoter methylation of RASSF1A. Representative methylated and un-methylated CpG sites in promoter region of all three genes are shown in Figure 2. Methylation density of each normal and adenomatous parathyroid tissue samples for all the three genes are shown as individual values in the scatter plot (Fig. 3). Correlation analysis of promoter methylation of all the genes with the disease parameters were summarized in the Table 3 and Supplementary Figure S2.

**Table 3:** Correlation analysis between promoter methylation status of genes and the disease parameters.

	CDKN2A methylation	CDKN2B methylation	RASSF1A methylation
Calcium	−0.18 (p = 0.68)	−0.23 (p = 0.54)	**−0.54 (p** = **0.02)**
PTH	−0.14 (p = 0.73)	0.48 (p = 0.17)	−0.15, (p = 0.5)
Phosphorus	−0.05 (p = 0.94)	−0.44 (p = 0.27)	−0.19 (p = 0.4)
ALP	0.38 (p = 0.36)	−0.18 (p = 0.7)	0.17 (p = 0.48)
25(OH)D	0.53 (p = 0.07)	0.01 (p = 0.98)	−0.31 (p = 0.22)
Tumor weight	**−0.48 (p** = **0.046)**	0.36 (p = 0.12)	−0.19, (p = 0.4)
Age	0.34 (p = 0.37)	−0.01 (p = 0.97)	−0.36 (p = 0.1)
Gender	0.12 (p = 0.57)	−0.31 (p = 0.14)	0.29 (p = 0.15)

**Figure 4:**
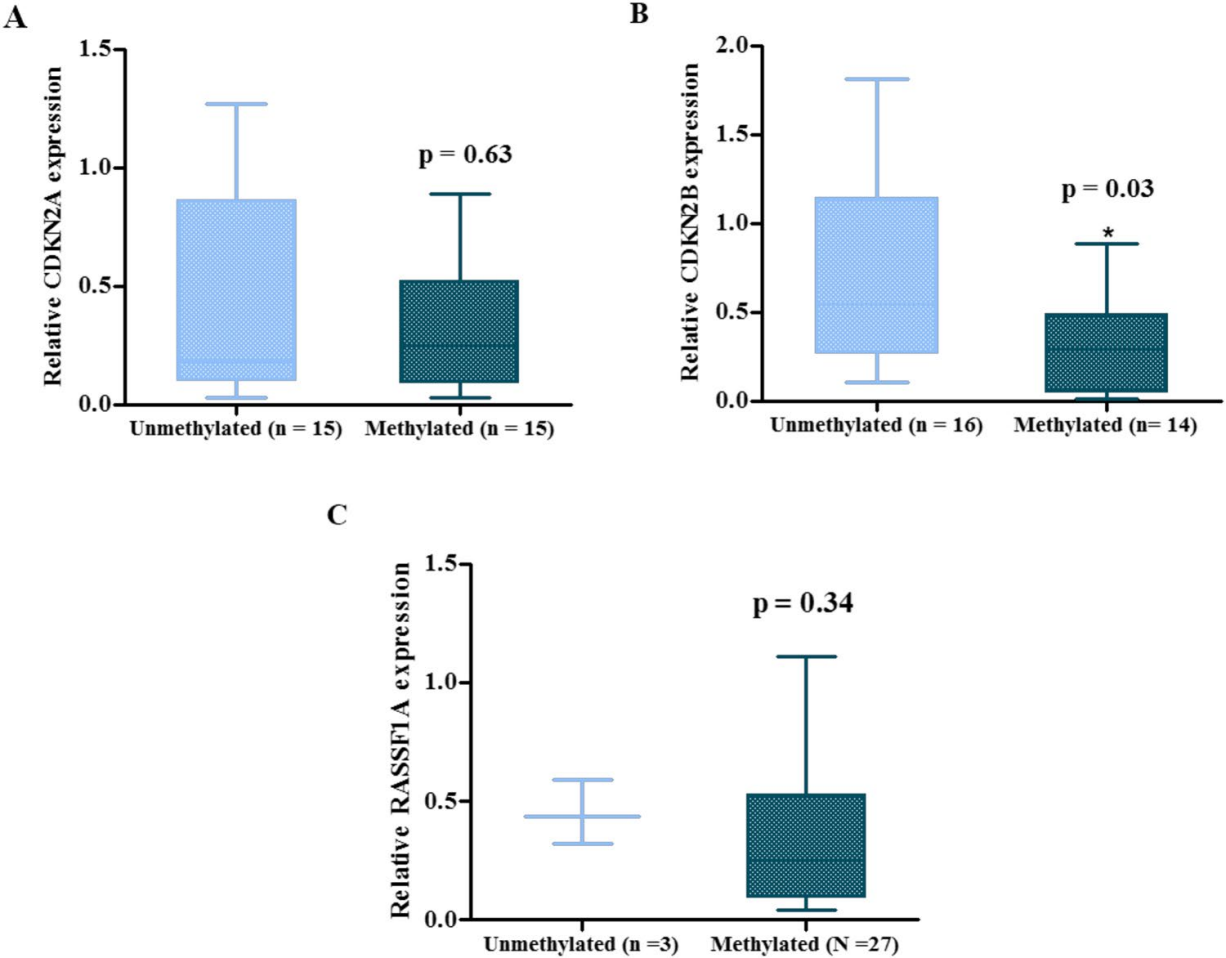
Comparison of relative gene expression level of (**A**) CDKN2A, (**B**) CDKN2B and (**C**) RASSF1A promoter methylation status determined using BSP. Box and whisker plot for comparative gene expression change between methylated and un-methylated tumor samples. The horizontal bars show the median values for the relative expression of in each group.

Further, expression patterns were compared between un-methylated and methylated tumor samples for all three genes. No difference was observed in CDKN2A expression levels between methylated and un-methylated group (0.33 ± 0.29 vs 0.42 ± 0.49, p = 0.63) (Fig. 4A). CDKN2B expression level was significantly low in methylated group compared to the un-methylated group (0.29 ± 0.27 vs 0.72 ± 0.56, p = 0.03) (Fig. 4B). RASSF1A expression also did not change between methylated and un-methylated patient groups (0.37 ± 0.35 vs 0.45 ± 0.13, p = 0.34) (Fig. 4C).

## Discussion

In this study we found that the promoter region of the three genes (CDKN2A, CDKN2B and RASSF1A) examined in sporadic parathyroid adenomas is frequently hypermethylated with the resultant reduced expression of their respective gene products. In addition, we have demonstrated an inverse relationship between expressions of the CDK inhibitors and cyclin D1 in the adenomatous compared to the normal parathyroid tissue. To the best our knowledge this is the first study to demonstrate hypermethylation of both the CDK inhibitors and point towards dysregulated cell cycle mechanisms in parathyroid adenomas.

In contrast to previous studies^12–14^, we found hypermethylation of cyclin dependent kinase inhibitors - CDKN2A and CDKN2B in about half of the adenomas. Starker *et al*.^14^ found that both CDKN2A and CDKN2B were inactivated by promoter hypermethylation, but others have not^12, 13^. No deletions or mutations were identified in CDKN2A and CDKN2B genes in parathyroid adenomas^11^. Transcriptional silencing due to hypermethylation of CDKN2A is one of the major mechanisms involved in the pathogenesis of several types of malignancies such as B cell lymphoma, hepatocellular, lung and colorectal cancers^21–23^. However, hypermethylation of CDKN2B has not been frequently reported in cancers. CDKN2A and CDKN2B encode p16 and p15 proteins respectively, that have important roles in the retinoblastoma pathway. Our study confirms the methylation-mediated transcriptional silencing of both CDKN2A and CDKN2B, and is the first study to demonstrate that the hypermethylation of CDKN2B is almost equivalent to CDKN2A in parathyroid adenomas. Thus, it appears that CDKN2B has an equivalent role as CDKN2A in parathyroid tumorigenesis. Based on our previous study and the results from the present study that demonstrated a positive association between CCND1 expression and adenoma weight^5^, we speculate that under-expression of tumor suppressor gene products (p15 and p16) and overexpression of oncogene (cyclin D1) may explain both the mechanism of parathyroid tumorigenesis and the severity of disease expression (higher adenoma weight and PTH level, and a more severe and symptomatic form of PHPT)^7^. Also, our study shows that the transcriptional silencing of CDKN2A and CDKN2B due to hypermethylation in at least 50% of sporadic parathyroid tumors. Although the expression was reduced in approximately 85% of the adenomas, hypermethylation of CKDN2A and CDKN2B promoter regions was found in a much smaller proportion implying that other mechanism may be involved in CDKN2A and CDKN2Breduced expressions.

The RASSF1A promoter region was hypermethylated in 90% parathyroid adenomas, and the methylation densities ranged from 20–100%. Three patients with no aberrant methylation had severe hypercalcemia (albumin-corrected serum calcium levels 14.6, 13.7 and 14.3 mg/dl). Both the reduced expression and hypermethylation were negatively associated with serum calcium levels of the PHPT patients, suggesting that patients with mild hypercalcemia had a significant reduction in expression and higher DNA methylation level of RASSF1A. This strongly suggests a role of hypermethylation-mediated RASSF1A tumor suppressor gene inactivation in parathyroid tumor formation and tumor progression. Similarly, Juhlin *et al*.^12^ have also reported reduced expression of RASSF1A with significant and frequent hypermethylation in parathyroid adenomas. Also Sulaiman *et al*. have reported hypermethylation of RASSF1A in 52% of PHPT patients with methylation densities ranging from 20–79%^13^. Thus, we suggest that promoter DNA methylation is responsible for the reduced expression of RASSF1A in parathyroid adenomas. RASSF1A encodes a Ras binding protein that has a role in the promotion of apoptosis, cell cycle arrest and genomic stability^24^. Mutations in RASSF1A have been only rarely reported, though hypermethylation-mediated inactivation is a major mechanism of regulating gene expression. RASSF1A inactivation by methylation has been observed in many cancers^25–27^ including endocrine tumors like pheochromacytoma and follicular thyroid cancer^28, 29^. There was no association between the methylation levels of each gene with the expression of cyclin D1.

Previous studies have identified that three tumor suppressor genes (RIZ1, SFRP1, HIC1) may be epigenetically-silenced in parathyroid adenomas^13, 14, 17^. We observed that the vitamin D receptor (VDR) and calcium sensing receptor (CASR) were not epigenetically silenced in parathyroid adenoma^15^. The inhibitory mechanism related to the effect of promoter methylation on transcription silencing is either by non-binding of transcription factors to methylated CpG sites or via binding of methyl-CpG binding domain (MBD) protein to methylated CpG sites irrespective of the sequence. These protein contains a transcriptional repression domain (TRD), which forms a complex with co-repressor molecules and histone deacetylase proteins (e.g., HDAC1, HDAC2). Then, this protein complex binds to methylated DNA and deacetylate the histones in chromatin. This in turn appears to condense the chromatin structure, making it more condensed and the DNA less accessible for transcription^30^.

The major limitations of our study was relatively small sample size and small number of normal parathyroid samples which needs to be confirmed on large sample size with variable hypercalcemic patients, and further validated at the protein level and functionally characterized by *in-vitro* experiments in parathyroid cells.

In conclusion, reduced expression of CDKN2A, CDKN2B and RASSF1A with significant hypermethylation of promoter region of these genes and inactivation of these genes through epigenetic regulation may be important factor in tumorigenesis in substantial proportion of parathyroid adenomas. Our study confirms previous observations on the importance of RASSF1A silencing by promoter hypermethylation in parathyroid tumorigenesis regardless of ethnicity. Overall the study provides better insights of highly deregulated cell regulatory mechanism in symptomatic PHPT.

## Methods

### Ethical approval

The research design was approved by the Institutional Ethics committee, Postgraduate Institute of Medical Education and Research (PGIMER), Chandigarh India. Informed consent was obtained from all study participants. The study was carried out in accordance with the approved guidelines.

### Subjects

Patients with PHPT were recruited from department of Endocrinology and General Surgery, Postgraduate Institute of Medical Education & Research, Chandigarh, India from January 2014 to December 2015. Parathyroid adenoma tissue samples (n = 30) were collected after parathyroidectomy and normal parathyroid tissue samples (n = 5) were obtained during elective thyroidectomy for multinodular goitre during which the normal parathyroid glands were removed inadvertently and stored at −80 °C. PHPT was diagnosed on the basis of presence of hypercalcemia and elevated PTH levels, and verified at surgery. After surgery, the diagnosis of parathyroid adenoma was confirmed by histopathology on hematoxylin and eosin (H&E) stained sections. The adenoma was encapsulated tumor with no fat and highly vascular. Patients with parathyroid hyperplasia, parathyroid carcinoma, secondary hyperparathyroidism, and multiple endocrine neoplasia syndromes were excluded.

### Expression analysis of CDKN2A, CDKN2B and RASSF1A genes

Tissue samples were collected in RNA *later* (Sigma-aldrich, USA) and stored at −80 °C and further used for RNA and DNA isolation. Total RNA was isolated from both adenoma and normal parathyroid tissue samples using Trizol method. The 260:280 nm ratio of RNA (interval 1.9–2.1) was determined by Biophotometer plus (Eppendorf, Hamburg, Germany) and confirmed by denaturing gel electrophoresis. cDNA was synthesized from 5µg total RNA (100µl reaction) by using iScript cDNA synthesis kit (Bio-Rad, USA) according to the manufacturer’s instructions.

Gene expression analysis was performed by quantitative real time PCR (qRT-PCR) for CDKN2A, CDKN2B, CCND1 and RASSF1A genes. Experiment was performed on ABI StepOnePlus real-time PCR system (Applied Biosystems, USA) as per the manufacturer’s recommendations using 18s rRNA as endogenous housekeeping gene. Primers for real time PCR were designed by using primer blast program (www.ncbi.nlm.nih.gov/tools/primer-blast/ *)*. For CDKN2A, forward primer 5′-ATATGCCTTCCCCCACTACC-3′ and reverse primer5′-CACATGAATGTGCGCTTAGG-3′; CDKN2B forward primer 5′-GAATGCGCGAGGAGAACAA-3′ and reverse primer 5′-CATCATCATGACCTGGATCGC-3′; RASSF1A forward primer 5′-TTACCTGCCCAAGGATGCTG-3′ reverse primer 5′-CAAGTACACTTGGCCGTGAC-3′; CCND1 forward primer 5′-TGTGCCACAGATGTGAAGTT-3′ and reverse primer 5′-CCGGGTCACACTTGATCACT-3′ and 18s rRNA forward primer 5′-GTAACCCGTTGAACCCCATT-3′ and reverse primer 5′-CCATCCAATCGGTAGTAGCG-3′ were used for amplifying the genes.

PCR reactions were carried out in a 96-well optical reaction plate. One µl of template cDNA (equivalent to 200 ng of total RNA) were added to 10 μl of PCR reaction mixture containing 0.25 μM each forward and reverse primers. Initial denaturation at 95 °C for 10 minutes then 40 cycles of 95 °C for 10 seconds, at different temperatures for 45 seconds and 72 °C for 45 seconds. All experiments were carried out in duplicate with two non-template controls as negative control. All samples were normalized to their corresponding housekeeping gene, 18s rRNA value and there after the normal parathyroid mean for each experiment. Finally, amplified products were sequenced (ABI 3730 sequencer, Applied Biosystems, USA) to confirm the correct transcript. Analysis of relative gene expression was calculated by the 2^−∆∆CT^ method to produce the data as fold change up/down regulation^31^.

### DNA extraction and bisulfite conversion

Total genomic DNA was extracted from both adenoma and normal parathyroid tissue samples by QIAmp mini DNA extraction kit (Qiagen, USA) according to the manufacturer’s instructions. DNA samples were subjected to bisulfite conversion using the EZ DNA Methylation-Gold^TM^ Kit (Zymo Research Corporation, CA, USA) involving chemical modification of unmethylated cytosine to uracil bases which are detected as thymine following PCR, but those that are methylated are resistant to this modification and remain as cytosine. After bisulfite conversion modified DNA was stored at −20 °C.

### Bisulfite-Sequencing PCR (BSP)

Methylation of CpG rich 5′ regions in CDKN2A, CDKN2B and RASSF1A genes were assessed by BSP using bisulfite sequencing primers designed by online program MethPrimer (http://www.urogene.org/methprimer/index1.html). For CDKN2A, forward primer 5′-TTTTTGAAAATTAAGGGTTGAGGGG-3′ and reverse primer 5′-AAAAAAACTAAACTCCTCCCCACCTAC-3′; for CDKN2B forward primer 5′-TTTATTGGGGATTAGGAGTTGAG-3′ and reverse primer 5′-CTAACAAAATAAAAAACCAACC-3′ and for RASSF1A forward primer 5′-GGGTTTTATAGTTTTTGTATTTAGGTTTTT-3′ and reverse primer 5′-AACTCAATAAACTCAAACTCCCCC-3′ were used for amplifying specific promoter region. Initial denaturation was performed at 95 °C for 1 minutes, followed by 40 cycles of PCR amplification (95 °C for 30 seconds, 60 °C for 30 seconds, and 72 °C for 45 seconds), and the final step was extended to 7 minutes at 72 °C for all three genes. Completely methylated by M.Sssl methyltransferase (New England Biolabs, USA) genomic DNA from a healthy individual was used as the positive control for methylation. After PCR, products were separated electrophoretically in 2% agarose gel and finally the amplified products were sequenced (ABI 3700, Applied Biosystems, USA). Sequencing data were further analysed for quantitation of percent methylation in promoter region by using BISMA (Bisulfite sequencing and DNA Methylation analysis) program^32^.

### Statistical Analyses

All statistical analyses were performed using Statistical Package for the Social Sciences version 20 (SPSS v20, IBM USA). Continuous (parametric) variables were presented as mean ± SD and non-parametric variables were also presented with geometric mean. Comparison of gene expression as well as DNA promoter methylation pattern between parathyroid adenoma and parathyroid normal (control) samples was analysed by Mann-Whitney U test or unpaired T-test. Mann-Whitney test was used to evaluate the differences in the relative expression level between the methylated and un-methylated group. Statistical tests were analysed with Bonferroni correction. Spearman’s rank correlation test and linear regression analysis was used for the correlation analyses of gene expression as well as DNA promoter methylation with the disease parameters (PTH, calcium, phosphorus, ALP, 25(OH)D, age and gender) as covariates. *p* value < 0.05 was considered statistically significant for all of our analyses.

### Data availability

Data generated or analysed during this study are included in this published article (and its Supplementary Information file). Additional data will be available from the corresponding author on reasonable request.

## Electronic supplementary material

Supplementary Information
