# Supplementary material for: Promoter hypermethylation inactivates CDKN2A, CDKN2B and RASSF1A genes in sporadic parathyroid adenomas
Source: Sci Rep. 2017 Jun 9;7:3123. doi: 10.1038/s41598-017-03143-8 (PMC5466668; doi:10.1038/s41598-017-03143-8)
Supplement: Supplementary file 1 — Supplementary Information [file 41598_2017_3143_MOESM1_ESM.doc]

**SUPPLEMENTARY INFORMATION**

**Promoter hypermethylation inactivates CDKN2A, CDKN2B and RASSF1A genes in sporadic parathyroid adenomas**

Ashutosh Kumar Arya^1^, Sanjay Kumar Bhadada^1^, Priyanka Singh^1^, Naresh Sachdeva^1^, Uma Nahar Saikia^2^, Divya Dahiya^3^, Arunanshu Behera^3^, Anil Bhansali^1^, Sudhaker D. Rao^4^

**Departments of Endocrinology, Postgraduate Institute of Medical Education & Research (PGIMER), Chandigarh India**

Ashutosh Kumar Arya, Sanjay Kumar Bhadada, Priyanka Singh, Naresh Sachdeva, Anil Bhansali

**Departments of Histopathology, Postgraduate Institute of Medical Education & Research (PGIMER), Chandigarh India**

Uma Nahar Saikia

**Departments of General Surgery, Postgraduate Institute of Medical Education & Research (PGIMER), Chandigarh India**

Divya Dahiya, Arunanshu Behera

**Bone & Mineral Research Laboratory, Henry Ford Hospital, Detroit USA**

Sudhaker D Rao

**Supplementary Figure S1:** Scatter plot showing the correlation of relative gene expression with disease parameters.

**Supplementary Figure S2:** Scatter plot showing the correlation of DNA promoter methylation with disease parameters

**Supplementary Figure S3: No aberrant DNA promoter methylation of CCND1 gene was observed in parathyroid adenoma**. After bisulfite conversion of genomic DNA, promoter region of CCND1 gene (nucleotide -386 to -4, from transcription start site) with product size of 383 bp was amplified and then sequenced. Representative chromatograms showing CpG sites in the promoter region of CCND1 gene in (A) parathyroid adenoma and (B) parathyroid control**.**

**A**

**
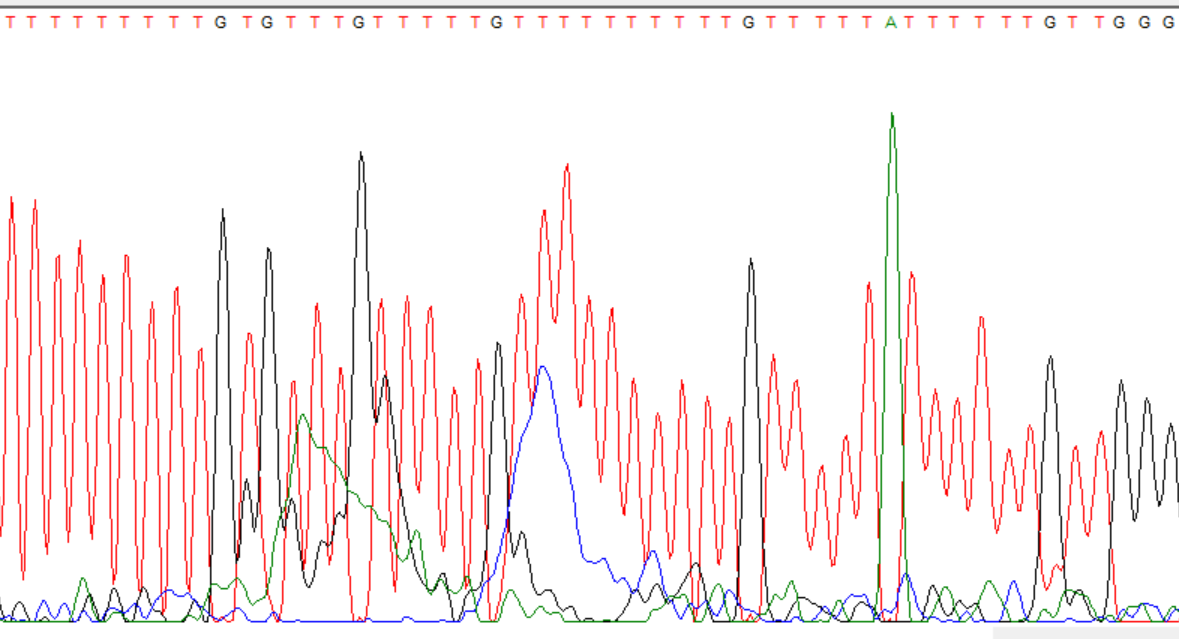
**

**B**

**
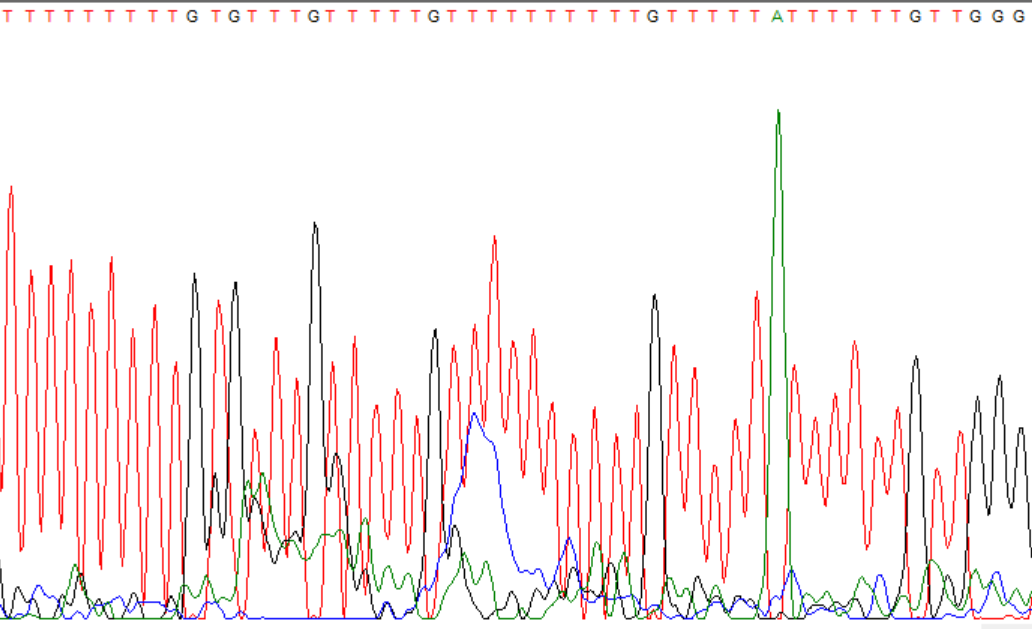
**
